# Digital Health Interventions for Cardiometabolic Health Outcomes in Rural and Remote Australia: A Systematic Review

**DOI:** 10.1111/ajr.70130

**Published:** 2025-12-26

**Authors:** Cheru Tesema Leshargie, Meless Gebrie Bore, Hazel Dalton, Subash Thapa, Fentaw Tadese Berhe, Zemenu Yohannes Kassa, Zekariyas Sahile Nezenega, Setognal B. Aychiluhm, Feleke Hailemichael Astawesegn, Birhanu Wondimeneh Demissie, Tebikew Yeneabat Mengist, Kedir Y. Ahmed

**Affiliations:** ^1^ Rural Health Research Institute, Charles Sturt University Orange New South Wales Australia; ^2^ School of Medicine and Dentistry, Griffith University Gold Coast Queensland Australia; ^3^ College of Medicine and Health Sciences, Hawassa University Hawassa Ethiopia; ^4^ School of Nursing and Midwifery, Faculty of Health, University of Technology Sydney Sydney New South Wales Australia; ^5^ Translational Health Research Institute, Western Sydney University, Campbelltown Campus Sydney New South Wales Australia

**Keywords:** cardiometabolic outcomes, digital health interventions, rural and remote Australia, systematic review, telehealth, telemedicine

## Abstract

**Introduction:**

Cardiometabolic disease contributes to increased morbidity and mortality in rural and remote Australia. Digital health technologies offer a promising solution to enhance healthcare access and support self‐management.

**Objective:**

This systematic review examined the effectiveness, feasibility and acceptability of implementing digital health interventions to improve cardiometabolic health outcomes in rural and remote Australia.

**Design:**

PubMed, MEDLINE, Embase, Scopus and CINAHL were searched from inception to end of July 2025. Eligible studies included interventional, observational and qualitative studies focused on digital interventions for cardiometabolic conditions. Due to heterogeneity among studies, a meta‐analysis was not conducted; instead, a narrative synthesis was used to summarise outcomes.

**Findings:**

Seventeen studies (7 RCTs, 1 quasi‐experimental, 7 observational and 2 qualitative) evaluated digital health interventions including video consultations, telephone coaching, apps, wearables and web platforms. Telemonitoring significantly reduced HbA1c (MD = −5.5%), with modest reduction via telephone support (RR = 0.96). Telestroke programs were associated with lower stroke mortality at 6 months (HR = 0.53) and 12 months (HR = 0.58). The review also demonstrated the feasibility and acceptability of digital health interventions, particularly when culturally tailored and delivered by local providers, with successful remote adaptation and high initial engagement. Interventions such as tele‐endocrinology and the “Healthy Weight” program were cost‐effective, contributing to improved HbA1c and quality of life. However, challenges included limited physical assessments, technical barriers and declining patient engagement over time.

**Conclusions:**

Digital health technologies, ranging from telehealth to mobile and web‐based tools, can enhance cardiometabolic outcomes in rural and remote settings, though barriers such as technology access and sustained engagement remain.

## Abstract (highlights)

**Key Points:**

- This systematic review synthesises evidence on digital health interventions for cardiometabolic health outcomes in rural and remote Australia, highlighting their growing role in chronic disease care.
- Digital interventions including telehealth, mobile apps, remote monitoring systems and web‐based programs improved self‐management, lifestyle behaviours and selected clinical outcomes such as blood pressure and glycaemic control.
- Interventions that incorporated remote monitoring with timely clinician feedback demonstrated the most consistent benefits across cardiometabolic indicators.
- Implementation challenges were common, particularly digital literacy barriers, limited internet connectivity and issues related to sustainability and integration into routine care in rural settings.
- Findings underscore the need for scalable, culturally appropriate and equity‐focused digital health models to strengthen cardiometabolic care and inform future policy, practice and research in geographically isolated communities.

**What Is Already Known on This Subject:**

- Cardiometabolic diseases (e.g., diabetes, cardiovascular disease, hypertension/stroke) are more prevalent and severe in rural and remote areas of Australia due to limited access to healthcare services.
- Digital health interventions (DHIs) like telehealth and mobile apps are increasingly used to improve healthcare delivery and self‐management in underserved populations.
- The effectiveness and implementation of DHIs in rural settings remain inconsistent, with known barriers including digital literacy, internet access and infrastructure limitations.

**What This Paper Adds:**

- This is the first systematic review to comprehensively assess both the effectiveness and implementation characteristics (feasibility, acceptability, cost‐effectiveness) of DHIs for cardiometabolic outcomes in rural and remote Australia.
- Telemonitoring interventions were associated with significant reductions in HbA1c, and telestroke models reduced mortality, indicating strong clinical impact.
- Culturally tailored, locally delivered interventions improved acceptability and trust, particularly in Indigenous and rural communities.
- DHIs showed economic benefits by reducing hospital admissions and healthcare costs, although sustained engagement and equity in access remain challenges.

**Abbreviations**
BMI: Body Mass Index
CI: Confidence Interval
CR: Cardiac Rehabilitation
CVDs: Cardiovascular Diseases
DALY: Disability‐Adjusted Life Years
DHIs: Digital Health Interventions
DID: Difference In Difference
DM: Diabetes Mellitus
HbA1c: Glycated Hemoglobin
HR: Hazard Ratio
MMM: Modified Monash Model
PRISMA: Preferred Reporting Items for Systematic Reviews and Meta Analyses
RCTs: Randomized Controlled Trials
ROBINS‐I: Risk Of Bias In Non‐randomized Studies of Interventions
SMS: Short Message Service
WHO: World Health Organization

## 1 Introduction

Cardiometabolic diseases such as cardiovascular diseases (CVD), diabetes mellitus (DM) and hypertension are the major public health concerns in today's society [1, 2]. The World Health Organization (WHO) estimated that CVD alone is responsible for an estimated 17.9 million deaths globally each year, while DM contributes to another 1.5 million [3, 4, 5, 6]. Beyond mortality, these conditions lead to long‐term complications, including disability and increased healthcare costs for individuals, families and societies [7, 8]. In 2019, nearly 400 million disability‐adjusted life years (DALYs) were lost due to CVD, a 24% increase from 316.9 million DALYs in 2000 [9]. Furthermore, these conditions place a substantial burden on healthcare systems and negatively impact patients' ability to work, overall quality of life and the well‐being of their families [10].

In Australia, CVD and DM also contribute to a substantial health burden, being the leading causes of both morbidity and mortality [11]. For example, CVDs (heart and blood vessel diseases and stroke) affect one in six Australians [12, 13] and are responsible for 30% of all deaths in the country [12, 13]. The burden is disproportionately higher in rural and remote areas of Australia compared to major cities [14], largely due to geographic isolation, low population density with limited access to specialist services and higher healthcare delivery costs in these regions [15, 16].

Digital health technologies (DHIs) are increasingly recognised as effective solutions to address healthcare disparities in rural and remote communities [17, 18]. These tools, such as telemedicine, mobile apps and wearable devices, can bridge access gaps by enabling remote health monitoring, virtual consultations and timely medical advice without the need for travel [19, 20]. The Australian federal and state governments have implemented various digital health initiatives to enhance access to medical services for individuals in rural and remote areas. For instance, the ‘Australian National Digital Health Strategy’ was developed to enhance healthcare accessibility for people living in rural and remote regions [21]. Similarly, the Queensland government introduced the ‘Digital Strategy for Rural and Remote, and Regional Healthcare’ to enhance improving healthcare access and support better health outcomes for rural and remote Queenslanders [18].

Despite the growing use of DHIs, their effectiveness, accessibility and appropriateness in rural Australia remain uncertain due to inconsistent implementation, digital literacy gaps and limited internet connectivity [22, 23]. Many rural communities face persistent digital divides that limit the reach and sustained impact of these technologies [23]. Moreover, the evaluation of these interventions is often inconsistent, making it difficult to determine their real‐world impact [24]. A comprehensive review is therefore essential to identify evidence‐based approaches, understand implementation barriers and enablers to inform policy [22]. This systematic review examined the effectiveness, feasibility and acceptability of implementing digital health interventions to improve cardiometabolic health outcomes in rural and remote Australia.

## 2 Methods

### 2.1 Study Designs

This systematic review was conducted following the Preferred Reporting Items for Systematic Reviews and Meta‐Analyses (PRISMA) guidelines [25], (File S1). The review synthesised quantitative and qualitative evidence on the impact of DHIs on cardiometabolic health outcomes in rural, remote and regional Australia. The review protocol was registered with the International Prospective Register of Systematic Reviews (PROSPERO) under the registration number of CRD42024623544.

### 2.2 Eligibility Criteria

This review included interventional (e.g., randomised controlled trials, quasi‐experimental), observational studies (e.g., cohort, cross‐sectional) and qualitative studies that assessed the implementation or effectiveness of DHIs for cardiometabolic outcomes. Eligible studies focused on conditions such as DM, CVD, obesity, hypertension, or stroke and were conducted in rural, remote, or regional Australia. Both community and facility‐based interventions were eligible. Studies were excluded if they focused solely on protocol development, did not report relevant cardiometabolic outcomes, were conducted exclusively in major Australian cities, or failed to report results separately for rural or remote settings. Systematic reviews, opinion pieces and purely descriptive studies without intervention evaluation were also excluded.

### 2.3 Operational Definitions

For the purposes of this review, digital health interventions (DHIs) were defined as the use of digital technologies, including telehealth (e.g., video consultations), mobile health (mHealth) apps, SMS‐based support, wearable monitoring devices, telemonitoring platforms and web‐based tools—aimed at improving health outcomes through prevention, monitoring, or treatment. DHIs were further categorised into self‐help tools, telehealth, and remote monitoring.

### 2.4 Self‐Help Tools

Interventions were primarily designed for individual use without real‐time clinician involvement. These included mobile apps, web‐based programmes, or SMS platforms used for self‐monitoring, education, lifestyle modification, or behaviour tracking.

### 2.5 Telehealth

Real‐time, synchronous interactions with healthcare providers using technologies such as video or telephone consultations.

### 2.6 Remote Monitoring

Use of wearable or home‐based devices that collect and transmit health data to providers for review, often with asynchronous feedback. These included integrated models involving interventions combining multiple components (e.g., teleconsultation, remote monitoring and behaviour change tools) into coordinated care frameworks.

Cardiometabolic health outcomes were operationally defined as clinical or behavioural measures related to DM, CVD, hypertension, stroke and obesity. These included outcomes such as HbA1c, blood pressure, BMI, lipid profile and self‐reported or device‐measured physical activity or dietary behaviours.

Rural and remote regions were defined based on the Australian Modified Monash Model (MMM) classification system, specifically including studies conducted in MM 2–7 areas and excluding those in MM 1 (Major cities) [26].

### 2.7 Outcome Measures

The primary outcomes of this review were major cardiometabolic conditions, specifically diabetes mellitus (DM), cardiovascular disease (CVD), obesity, hypertension and stroke. These outcomes were assessed as reported by included studies. Clinical measures included validated indicators such as glycated haemoglobin (HbA1c) for DM management, blood pressure levels for hypertension control and BMI for obesity assessment [27, 28]. Cardiovascular‐ and stroke‐related outcomes were extracted where available [29]. In addition, implementation outcomes, such as acceptability, feasibility, cost and sustainability of digital health interventions in rural and remote settings were considered where reported.

### 2.8 Information Sources and Search Strategy

A comprehensive literature search was conducted across five electronic databases (PubMed, MEDLINE, EMBASE, Scopus and CINAHL) using a combination of standard terms and free‐text keywords. The reference lists of all included articles were also checked to identify additional eligible studies. The search was conducted in two stages: an initial search in December 2024, followed by an updated search in July 2025 to capture any newly published studies. A medical research librarian ensured search comprehensiveness and reproducibility. All retrieved records were imported into Covidence for deduplication and screening. Detailed search strategies for all databases are provided as File S2.

### 2.9 Study Selection Process

**FIGURE 1:**
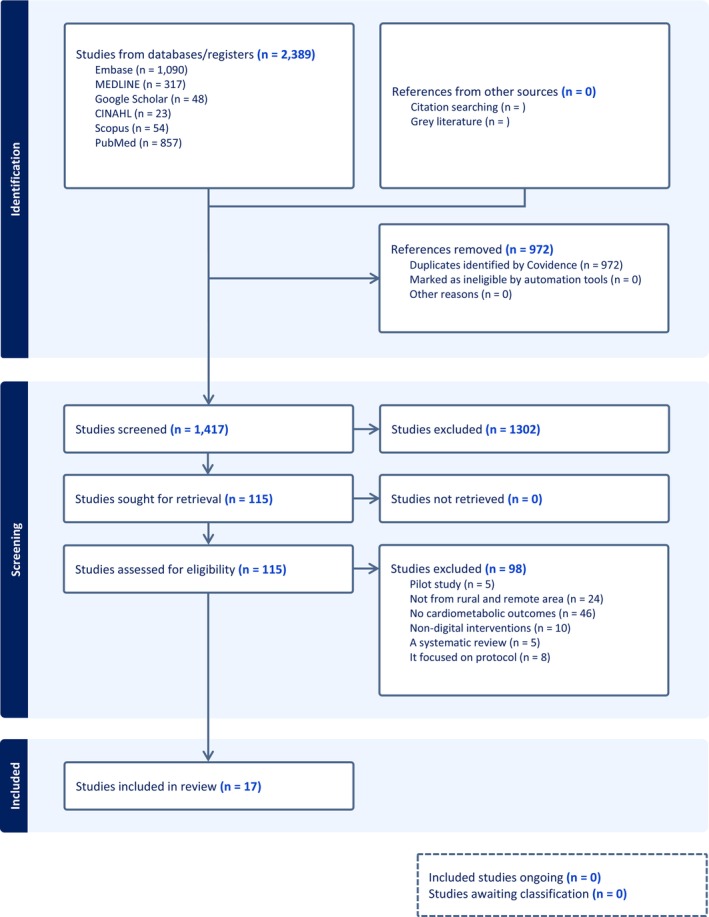
PRISMA flow diagram.

All identified studies were imported into EndNote (v21.5) [30] for reference management and then subsequently exported to Covidence for systematic screening and selection. Two independent reviewers (CTL and MGB) screened all articles based on predefined inclusion and exclusion criteria. Initially, screening of titles and abstracts was carried out to identify studies eligible for full‐text review. Subsequently, full‐text screening was then undertaken for articles that met the inclusion criteria during the title and abstract screenings. Any disagreements between reviewers were resolved through discussion, and if a consensus was not reached, a third reviewer, KYA, provided arbitration. Excluded studies were documented with reasons for exclusion. To ensure transparency and reproducibility, the study selection process is presented in a PRISMA flow diagram (Figure 1), outlining the number of records identified, screened, excluded and included in the final analysis.

### 2.10 Data Extraction and Data Items

Data extraction was conducted using a standardised form adapted from the Cochrane Handbook for Systematic Reviews of Interventions [31]. Extracted data included study characteristics (e.g., author(s), year, country, design, sample size, population), intervention details (e.g., type, duration, frequency, delivery method) and outcomes assessed (e.g., glycaemic control, cardiovascular markers, weight management, physical activity, dietary behaviour, psychological outcomes). Additional information on follow‐up periods, adherence and cost‐effectiveness was also recorded. Two independent reviewers (CTL and MGB) performed the extraction, resolving discrepancies through discussion or, if necessary, arbitration by a third reviewer (KYA).

Quantitative data extraction included means, odds ratios (OR), relative risks (RR), hazard ratios (HR), confidence intervals (CI) and *p*‐values, while qualitative data encompassed key themes, participant quotations and contextual factors. Where data were missing or unclear, corresponding authors were contacted via email, and up to two attempts were made to seek clarification. Data were managed using Microsoft Excel, with a summary table provided (provided in Files S3 and S4).

### 2.11 Risk of Bias Assessment

The risk of bias was assessed for all included studies using validated tools specific to each study design. For randomised controlled trials (RCTs), the Cochrane Risk of Bias 2.0 (RoB 2.0) tool was used, which assesses domains such as the randomisation process, deviations from intended interventions, missing outcome data, measurement of outcomes and selective reporting [32]. Non‐randomised experimental studies were assessed using the Risk Of Bias In Non‐randomised Studies of Interventions (ROBINS‐I) tool, focusing on potential confounding, participant selection, intervention classification, deviations from intended interventions, missing data, outcome measurement and selective reporting [33]. The Joanna Briggs Institute (JBI) Critical Appraisal Tools were applied to observational studies like observational and qualitative studies to evaluate methodological rigour, reliability and validity [34].

Two independent reviewers (CTL and MGB) conducted the risk of bias assessments. Discrepancies were resolved through discussion. The risk of bias assessment for the randomised controlled trials (RCTs) was categorised as ‘low risk’, ‘some concerns’, or ‘high risk of bias’ [32]. Non‐randomised studies of interventions were assessed as having ‘low risk of bias’, ‘moderate risk of bias’, ‘serious risk of bias’, or ‘critical risk of bias’ [35]. Furthermore, the methodological quality of observational studies was assessed using the Newcastle‐Ottawa Scale (NOS). Following established guidelines, studies scoring 7–9 were classified as high quality (low risk of bias), 4–6 as moderate quality and 0–3 as low quality (high risk of bias) [36].

### 2.12 Methods of Analysis

Descriptive statistics were used to summarise the characteristics of included studies, such as participant demographics, intervention types, outcomes, sample size and study design. Due to substantial heterogeneity in intervention types, study populations, follow‐up durations and outcome measures, conducting a meta‐analysis was not feasible. Therefore, a structured narrative synthesis approach was employed. A thematic narrative synthesis was conducted, with results grouped by cardiometabolic condition (e.g., DM, CVD, obesity, stroke, hypertension) and intervention type (e.g., telemonitoring, mobile apps, telehealth). This approach allowed for a comprehensive interpretation of the data while acknowledging variability in study designs and methodologies. Effect size measures were directly extracted from the studies as reported by the authors. These included comparisons between interventions and control groups, such as odds ratios (OR), differences in proportions between pre‐and post‐intervention outcomes and difference‐in‐difference (DID) measures. Where available, 95% confidence intervals (CIs) and *p*‐values were also obtained to assess the statistical significance of the results. For example, studies evaluating the impact of digital health interventions on DM management reported effect sizes such as ORs for improvements in HbA1c levels and frequency of blood glucose monitoring, alongside corresponding CIs and *p*‐values, providing insight into the interventions' effectiveness.

Findings were organised into different themes based on the cardiometabolic outcomes of interest, such as DM, CVD, obesity, hypertension and stroke. This approach facilitated the identification of patterns and trends across studies, even in the absence of a meta‐analysis. For instance, studies on weight management interventions consistently reported mean weight reductions and BMI changes, along with their 95% CIs, highlighting the effectiveness of digital technologies in promoting sustained weight loss. Additionally, qualitative insights from studies exploring participant experiences and implementation challenges were integrated into the synthesis, providing context to the quantitative findings.

### 2.13 Ethical Considerations

As this study involved secondary analysis of published literature, ethical approval was not applicable. However, as part of the data extraction process, we recorded whether each included study reported obtaining ethical approval from a relevant ethics committee or institutional review board. This was used as an indicator of ethical compliance. No further assessment of ethical procedures (e.g., consent processes or data protection) was conducted, as such information was often not reported in the primary studies.

## 3 Results

### 3.1 Overview of Included Studies

A total of 2389 records were identified through database searches. After removing 972 duplicates using Covidence, 1417records remained and were assessed during the title and abstract screening stage. This stage excluded 1302 records, leaving 117 studies for full‐text review. During full‐text screening, 98 studies were excluded for the following reasons: pilot studies (*n* = 5), not conducted in rural or remote areas (*n* = 24), absence of cardiometabolic outcomes (*n* = 46), non‐digital interventions (*n* = 10), systematic reviews (*n* = 5) and protocol‐only studies (*n* = 8). The final review included 17 studies (See Figure 1): seven RCTs [37, 38, 39, 40, 41, 42, 43], one quasi‐experimental [44], two qualitative [45, 46] and seven observational or mixed‐method studies [47, 48, 49, 50, 51, 52, 53]. Eight studies were conducted in Queensland [39, 40, 41, 44, 47, 49, 51, 52], four in NSW [37, 38, 42, 46], one in Western Australia [50], two in South Australia [48, 53], one in Victoria [43] and the remaining one was conducted in more than one state [45].

### 3.2 Types of Digital Health Interventions

The DHIs identified in this review were organised into four categories. Self‐help tools included mobile apps, digital self‐management platforms, SMS and mailed kits, enabling users to engage in behaviour change independently [37, 38, 39, 46, 52, 53]. Remote monitoring and tele‐tracking technologies comprised wearable devices and tools such as glucometers that supported ongoing health tracking and feedback [38, 41, 42, 51]. Telemedicine and tele‐prescription models covered video consultations, telephone‐based care and asynchronous communication like text messaging for clinical support and medication management [39, 40, 42, 44, 46, 47, 48, 49, 51, 53]. Lastly, integrated care models involved hybrid approaches combining digital and face‐to‐face services, often delivered through multidisciplinary teams to ensure coordinated and comprehensive care [51].

### 3.3 Types of Cardiometabolic Health Outcomes

Digital health interventions were implemented for the management of DM in eight studies [39, 40, 41, 44, 47, 49, 51, 52], CVD in seven studies [39, 42, 50, 54, 55, 56, 57], obesity in four studies [38, 39, 40, 42, 46] and hypertension and stroke in four studies [37, 40, 44, 48]. Additional outcome measures included psychological factors [37, 45], cost‐effectiveness metrics (e.g., intervention costs and healthcare savings) [41, 47, 49, 50, 53, 57] and access‐to‐care indicators such as follow‐up adherence and consultation frequency [45, 47, 49]. The duration of interventions ranged from 6 weeks to 96 weeks, with follow‐up periods capturing both short‐ and long‐term effects.

### 3.4 Risk of Bias and Quality of Included Studies

Among the seven RCTs included in this review, one (14.3%) [38] was rated as having a high risk, five (71.4%) [39, 40, 41, 42, 43], were rated as having some concerns and the remaining one (14.3%) [37], was rated as having a low risk of bias, see File S5. Additionally, a quasi‐experimental study by Millan et al. [44], was rated as having a serious risk of bias. Among the eight observational, mixed‐methods and qualitative studies included in this review, two (25%) were rated as high quality [45, 48], one (12.5%) as moderate to high quality [50], four (50%) as moderate quality [46, 47, 49, 53] and two (25%) as low to moderate quality [51, 52].

### 3.5 Digital Health Interventions for the Management of Diabetes Mellitus

A total of nine studies [39, 40, 41, 44, 45, 47, 49, 51, 52] investigated the impact of digital health on DM management in rural and remote Australia. Warren et al. [41] evaluated a telemonitoring program for type 2 DM in Townsville, Queensland, incorporating home monitoring devices for blood glucose and blood pressure, with support from a DM care coordinator. The intervention led to a significant reduction in HbA1c levels (8.4% to 7.5%, *p* = 0.004), while the control group showed no change (8.1%). Additionally, Eakin et al. [39] in a study implemented at Logan, Queensland, found that a digital health intervention delivered via telephone significantly improved glycaemic control, with an HbA1c reduction of −0.6% (*p* < 0.001).

William et al. [51] evaluated the effectiveness of a telehealth‐based paediatric DM care model in rural Queensland, where videoconferencing consultations between four rural hospitals and specialists at Mackay Base Hospital (MBH) were conducted. The telehealth group had lower HbA1c levels (7.5%) compared to the central clinic group (8.4%), suggesting non‐inferiority of telehealth care. Moreover, Mullan et al. [44], examined the impact of the Virtual Care Diabetes Education Program (VCDEP) on DM care documentation in Western Queensland. The intervention significantly improved HbA1c recording (*p* < 0.001), eGFR recording (*p* < 0.001), total cholesterol recording (*p* = 0.022) and foot examination documentation (*p* = 0.015). However, no significant changes were observed in achieving clinical DM targets, including blood pressure control (*p* = 0.205), HbA1c reduction (*p* = 0.306), eGFR improvement (*p* = 0.394) and BMI target achievement (*p* = 0.672).

Goode et al. [40], also reported that a telephone‐based digital health intervention in Logan, Queensland led to a significant reduction in HbA1c at 6 months in the high‐call group at the same place (RR = 0.96, 95% CI: 0.92–0.99, *p* = 0.02); however, no significant reduction was observed in medium‐ or low‐call groups. Beyond glycaemic control, digital health interventions have contributed to broader DM management. Fatehi et al. [47] evaluated telehealth for DM management in remote Queensland, where patients 210–1800 km from Brisbane accessed a tele‐endocrinology clinic. The intervention led to medication changes in 62% of consultations, with 75% involving lab tests and 86% scheduling follow‐ups. While improving access, 21% needed in‐person exams, 5% required follow‐ups and clinicians felt decisions could be better in 34% of cases if conducted in person.

Adu et al. [52], evaluated a mobile app for DM self‐management in North Queensland, reporting an 84% retention rate and high engagement, with 68% of users tracking blood glucose levels. Similarly, Graham et al. [45] demonstrated the value of digital health in managing diabetic foot disease among Aboriginal and Torres Strait Islander communities in rural South Australia and New South Wales by improving access to specialists and enhancing patient engagement. The intervention also fostered increased trust in care, with many patients expressing reassurance that specialists had directly assessed their condition.

### 3.6 Digital Health Interventions for the Management of Cardiovascular Diseases

Three studies assessed the role of digital interventions in improving CVD outcomes, highlighting their potential to improve rehabilitation, weight management, diagnostic efficiency and dietary adherence in rural and remote Australia [42, 50, 53]. Champion et al. [53] examined the impact of telehealth‐based cardiac rehabilitation (CR) programs in regional and remote South Australia, comparing in‐person vs. telehealth CR. During the COVID‐19 pandemic, CR attendance declined significantly (46.6% vs. 59.9%, *p* < 0.001) and completion rates were lower (42.4% vs. 75.4%, *p* < 0.001). However, telehealth‐based CR demonstrated higher attendance and completion rates than traditional in‐person programs, supporting the viability of remote delivery models for cardiac rehabilitation.

Sangster et al. [42] analysed a weight loss and quality of life intervention for CVD patients in urban and rural Australia. The Healthy Weight group achieved greater weight loss than the Physical Activity group (1.6 kg vs. 0.4 kg, *p* = 0.005), with 19% losing ≥ 5% of body weight. Quality of life also improved significantly (0.081 QALYs vs. 0.037 QALYs, *p* < 0.05). These studies emphasise the efficacy of digital interventions in CVD prevention and management, primarily highlighting their benefits for rural cardiac patients and those who did not attend CR programs.

In Western Australia, Tually et al. [50] implemented a telehealth‐enabled nuclear cardiology intervention in Kalgoorlie, using a web‐based telemedicine system for myocardial perfusion scintigraphy (MPS) imaging and remote consultations. The intervention led to treatment plan modification for 76% of patients, reducing unnecessary admissions and investigations. Furthermore, 50% of scans showed abnormal perfusion and 5% required urgent intervention.

### 3.7 Digital Health Interventions for the Management of Hypertension and Stroke Management

Our review identified two studies on digital health interventions for stroke and hypertension management. Goh et al. [48] evaluated a Telestroke program in regional and rural South Australia, incorporating secure video consultations, multimodal CT perfusion imaging and comprehensive stroke management plans. The intervention significantly reduced mortality at 6 months (HR = 0.53, 95% CI: 0.41–0.69, *p* < 0.001) and 12 months (HR = 0.58, 95% CI: 0.44–0.76, *p* < 0.001).

### 3.8 Digital Health Interventions for the Management of Obesity

Our review identified four studies [37, 38, 40, 43], on digital health interventions for obesity management. Duncan et al. [38] assessed a telehealth‐based program in Newcastle integrating a smartphone app, Fitbit tracking and calorie‐counting tools but found no significant weight changes at six or twelve months (*p* > 0.05). Additionally, Goode et al. [40] evaluated a telephone‐based lifestyle intervention for weight loss in primary care settings in Logan, Queensland. The high‐call group achieved significant weight loss at 6 months (−2.7 kg, *p* < 0.001), 18 months (−2.5 kg, *p* < 0.001) and 24 months (−2.0 kg, *p* = 0.002), while no significant changes were observed in the medium‐ and low‐call groups. Furthermore, Drew et al. [37] examined the SHED‐IT Recharge telehealth intervention for weight management, physical activity and sedentary behaviour in Newcastle, New South Wales. The program led to significant weight loss at 3 months (−4.2 kg, *p* < 0.001) and 6 months (−3.9 kg, *p* < 0.001).

Moreover, Lombard et al. [43] reported that participants who received the HeLP‐her lifestyle intervention, a low‐intensity, community‐based program involving one group session, monthly SMS reminders, a program manual and a single phone coaching session—experienced a mean weight change of −0.48 kg (95% CI: −0.99 to 0.03), compared to a mean weight gain of +0.44 kg (95% CI: −0.09 to 0.97) in the control group. The adjusted between‐group difference was −0.87 kg (95% CI: −1.62 to −0.13), indicating a statistically significant effect of the intervention (*p* = 0.02). Talay et al. [46] demonstrated that the Eucalyptus Digital Weight‐Loss Service, combining digital delivery with GLP‐1 receptor agonist support, was effective in achieving early and meaningful weight loss among regional Australians, enhancing confidence, exercise motivation and quality of life.

### 3.9 The Cost‐Effectiveness of Digital Health Interventions

Five of the sixteen studies in our review assessed the cost‐effectiveness of digital interventions [41, 47, 50, 51, 57]. Warren et al. [41] found that a telehealth intervention in primary care significantly reduced costs ($3781 vs. $4662, *p* < 0.001). Sangster et al. [57] showed that a digital weight loss program for cardiac patients was more cost‐effective ($1260 vs. $2112 per participant). Fatehi et al. [47] and William et al. [51] reported cost savings in DM management through video consultations, reducing travel burdens and improving access to care. Tually et al. [50] found that a telehealth‐enabled cardiology intervention reduced unnecessary admissions and investigations.

### 3.10 Feasibility and Acceptability of the Implementation

Feasibility and acceptability were consistently demonstrated across the included studies (*n = 17*), despite variations in intervention type and setting. Four studies successfully transitioned from in‐person to remote delivery during the COVID‐19 pandemic [37, 47, 51, 53]. Videoconferencing [47], online programs [37] and telehealth consultations (50, 52) were implemented with minimal disruption. High initial engagement was observed in studies using prompts and interactive features [37, 52], though sustaining this over time was challenging [38]. Acceptability was highest in culturally tailored interventions involving local health professionals, particularly in Indigenous and rural contexts [45, 51], where trust and cultural safety improved uptake. Key barriers included low digital literacy, poor internet access, manual data entry burdens and comorbid mental health issues [40, 47, 52, 53]. Successful implementation was supported by structured protocols, behaviour change techniques (e.g., SMART goals, CBT) and nurse‐led, standardised workflows [40, 44, 47, 48, 49], which improved fidelity. However, long‐term sustainability was variable, with some studies [37, 38] reporting declining effects after 6–12 months, highlighting the need for continued engagement strategies.

## 4 Discussion

To the best of our knowledge, this is the first review to comprehensively examine the implementation and clinical outcomes of DHIs on cardiometabolic disorders in rural and remote Australia. Our findings showed that telemonitoring and telephone‐based intervention programs were associated with significant reductions in HbA1c levels among patients with diabetes. The Telestroke program also significantly reduced stroke mortality among patients. Additionally, DHIs were found to be cost‐effective by lowering healthcare costs, reducing travel burdens and unnecessary admissions and improving access to care.

People living in Australia benefit from one of the world's highest life expectancies, supported by a robust healthcare system that includes Medicare, private health insurance, local health networks and government‐led initiatives [58, 59]. The system operates through coordinated efforts across federal, state and local levels to provide safe and affordable care [58, 60]. However, despite innovative aeromedical and other solutions (e.g., the Royal Flying Doctors Service) [61], disparities remain in rural and remote areas, where limited access to specialist services negatively impacts patient experiences, reduces care quality and contributes to poorer health outcomes [62, 63, 64]. This is especially important for chronic conditions such as DM, CVD and hypertension, which often require ongoing specialist care and advanced medical technologies [65]. The persistence of these disparities underscores the growing importance of DHIs such as telemedicine, mobile health apps, and remote monitoring tools to bridge gaps in service delivery and improve cardiometabolic health outcomes in underserved regions.

Our review showed that DHIs, including self‐help tools, remote monitoring and tele‐tracking technologies, telemedicine and tele‐prescription models and integrated care models, can support the Australian healthcare system by providing accessible remote care for managing cardiometabolic diseases [37, 39, 41, 44, 47, 48, 50, 51, 52, 53]. At the individual or household level, digital self‐help tools can assist with chronic disease management by enabling symptom monitoring, health‐metric tracking and access to educational resources. Global evidence from previous systematic reviews indicates that structured self‐management support in primary care, including self‐help materials, targeted follow‐up and shared goal setting can improve clinical outcomes, self‐efficacy, psychological healt and quality of life across diverse populations [66]. Findings from this study also demonstrated that self‐help tools contributed to improvements in cardiometabolic outcomes such as glycaemic control, weight loss and increased physical activity levels [37, 38, 39, 52].

Our review highlights the value of remote monitoring and tele‐tracking technologies in improving cardiometabolic outcomes in rural and remote Australia by enabling continuous monitoring of physiological parameters and behaviour patterns, and by providing timely data to both patients and healthcare providers [67]. Consistent with a recent systematic review, we also noted that remote monitoring systems and tele‐tracking technologies can improve clinicians' capacity (i.e., workforce upskilling) to manage chronic diseases by facilitating the early detection of warning signs, contributing to effective personalised care for DM and CVD [68], such as improvements in glycaemic control, HbA1c documentation, blood pressure monitoring and patient follow‐up adherence. These interventions, including home‐based monitoring devices and wearable technologies, were especially effective in geographically isolated communities by reducing clinical inertia, facilitating early intervention and supporting long‐term disease management through proactive, data‐driven care.

In addition to individualised monitoring, telemedicine and tele‐prescription models are instrumental in extending specialist care access to geographically isolated populations. Evidence demonstrates that telemedicine and tele‐prescription models play a critical role in enhancing healthcare access by facilitating virtual consultations, remote prescribing and the delivery of multidisciplinary care across vast geographic areas [19, 69]. Evidence from our review confirmed that such models were instrumental in managing conditions like DM, CVD, obesity, hypertension and stroke. Notable studies by Smith et al. [49], Tually et al. [50] and Goh et al. [48] showed that video and telephone‐based interventions improved clinical outcomes, reduced mortality, increased consultation rates and led to significant treatment modifications. These digital approaches enabled patients to receive timely expert care without the burden of travel, demonstrating that telemedicine is a feasible and effective model for addressing specialist care gaps in rural and remote Australia [70].

Evidence shows that integrated care models supported by DHIs enhance coordination between primary and specialist care, while improving the cost‐efficiency of healthcare delivery [71, 72]. Our findings highlight that these models contributed to reduced healthcare costs, optimised resource use and improved access to care in rural settings. Five studies [41, 42, 47, 50, 51], reported economic benefits ranging from decreased hospital admissions to lower per‐patient intervention costs. These interventions demonstrated that digitally enabled integrated care models can effectively deliver high‐value care for cardiometabolic conditions, ensuring both improved clinical outcomes and sustainability in underserved populations.

The review also identifies key challenges in implementing DHIs for CVD management in rural Australia, particularly regarding patient engagement and participation. Telehealth‐based cardiac rehabilitation [47] and weight management programs [38] demonstrated high levels of patient engagement, particularly when structured support and personalised guidance were provided. However, telehealth CR declined during the COVID‐19 pandemic [47]. Moreover, other contextual factors such as poor digital literacy, poor internet connectivity, lack of cultural appropriateness in content and the lack of integration of digital health within existing healthcare infrastructure hinder the effective implementation [73, 74]. Ongoing challenges also relate to sustaining engagement, ensuring cost‐efficiency and promoting equitable access.

## 5 Strengths and Limitations of the Study

### 5.1 Strengths

First, the review includes a wide range of study designs (RCTs, quasi‐experimental, observational and qualitative), offering a rich and nuanced understanding of how digital health interventions impact cardiometabolic outcomes in rural and remote settings. This diversity strengthens the external validity and real‐world relevance of the findings. By applying the MMM for rurality and operationally defining key concepts like DHIs and cardiometabolic outcomes, the review ensures consistent study selection and transparency, enhancing methodological rigour. The results are organised thematically by both disease category and intervention type (e.g., diabetes, CVD, obesity; telemonitoring, mobile apps), offering clear, actionable insights for health system stakeholders, rural health policymakers and digital health implementers.

### 5.2 Limitations

The wide variation in design, intervention types, outcomes and follow‐up periods precluded a meta‐analysis, preventing pooled effect estimates and formal cross‐study comparisons. Additionally, the geographic distribution of the included studies was uneven, with most conducted in Queensland and New South Wales and limited representation from regions such as the Northern Territory and Tasmania, potentially limiting the generalisability of the findings across all remote Australian contexts. Another limitation relates to the search strategy, which focused on peer‐reviewed literature and may have excluded relevant interventions implemented in rural and remote settings but not formally reported or published.

## 6 Conclusions

This review highlights the potential of DHIs to improve cardiometabolic outcomes in rural and remote Australia, with notable benefits for diabetes, hypertension, CVD and obesity. Telemonitoring was especially effective for DM and hypertension, while telehealth and videoconferencing enhanced access to specialist care and cardiovascular disease management. Text messaging and web‐based platforms showed promise for obesity prevention and structured weight loss support. However, challenges persist, including maintaining patient engagement, ensuring long‐term sustainability, cost‐effectiveness and equitable access. Future research should focus on refining intervention design, addressing digital literacy barriers, conducting rigorous economic evaluations and prioritising culturally safe and responsive approaches. As trust and engagement barriers were commonly identified across studies, embedding cultural safety into digital health interventions may enhance their acceptability and long‐term effectiveness, particularly among marginalised or underserved populations.

## Artificial Intelligence (AI) Usage Statement

Artificial intelligence (AI) usage statement: The authors declare that no generative artificial intelligence (AI) was used to create any content, ideas, or interpretations in this systematic review. AI was used only to edit grammar, clarity and readability across the entire manuscript. No AI tools were involved in study design, data extraction, analysis, interpretation, or conclusion development.

Role of the funder/sponsor: The funder had no role in the design of the systematic review; in the development of the search strategy; in the study selection, data extraction, quality appraisal, or data synthesis; or in the interpretation and reporting of the findings. The funder also had no role in the preparation, review, or approval of the manuscript, nor in the decision to submit the manuscript for publication.

## Author Contributions

**Cheru Tesema Leshargie:** conceptualisation, data curation, methodology, search strategy development, database searching, study screening, data extraction quality and risk – of – bias assessment, formal analysis, writing – original draft, visualisation, writing – review and editing, project administration, corresponding author. **Kedir Y. Ahmed:** conceptualisation, data curation, methodology, search strategy development, database searching, methodological guidance, validation, resolution of disagreements in screening, data extraction, and risk – of – bias assessment, writing – review and editing. **Meless Gebrie Bore:** data curation, study screening, data extraction, quality and risk – of – bias assessment, writing – review and editing. **Hazel Dalton, Subash Thapa, Fentaw Tadese Berhe, Zemenu Yohannes Kassa, Zekariyas Sahile Nezenega, Setognal B. Aychiluhm, Feleke Hailemichael Astawesegn, Birhanu Wondimeneh Demissie, and Tebikew Yeneabat Mengist:** writing – review and editing, critical revision of the manuscript. All authors approved the final version of the manuscript.

## Funding

This work was funded by a grant from the Commonwealth of Australia, represented by the Department of Health (Grant Activity 4‐DGEJZ1O/4‐CW7UT14).

## Consent

The authors have nothing to report.

## Conflicts of Interest

The authors declare no conflicts of interest.

## Supporting information


**File S1:** PRISMA 2020 checklist showing compliance with reporting guidelines for this systematic review.
**File S2:** Full electronic search strategies for included databases (PubMed, Embase, Ovid MEDLINE and CINAHL).
**File S3:** Characteristics of the included studies.
**File S4:** Describe summary of study characteristics and health outcomes from digital health intervention trials addressing cardiometabolic conditions in rural and remote settings.
**File S4:** Risk‐of‐bias assessment for all included randomised controlled trials across the five RoB 2 domains and overall bias rating.

## Data Availability

The data that support the findings of this study are available on request from the corresponding author. The data are not publicly available due to privacy or ethical restrictions.
