# Supplementary material for: Digital Health Interventions for Cardiometabolic Health Outcomes in Rural and Remote Australia: A Systematic Review
Source: Aust J Rural Health. 2025 Dec 26;33(6):e70130. doi: 10.1111/ajr.70130 (PMC12742273; doi:10.1111/ajr.70130)
Supplement: Supplementary file 1 — File S1: PRISMA 2020 checklist showing compliance with reporting guidelines for this systematic review. File S2: Full electronic search strategies for included databases (PubMed, Embase, Ovid MEDLINE and CINAHL). File S3: Characteristics of the included studies. File S4: Describe summary of study characteristics and health outcomes from digital health intervention trials addressing cardiometabolic conditions in rural and remote settings. File S4: Risk‐of‐bias assessment for all included randomised controlled trials across the five RoB 2 domains and overall bias rating. [file AJR-33-0-s001.zip › ajr70130-sup-0003-supinfo02.docx]

***Supplementary file 2:*** *Full Electronic Search Strategies for included Databases (PubMed, Embase, Ovid MEDLINE, and CINAHL).*

| **Database** | **Search Strategy** |
| --- | --- |
| **PubMed** | ("telehealth"[MeSH Terms] OR "telemedicine"[MeSH Terms] OR "mobile health"[Title/Abstract] OR "eHealth"[Title/Abstract] OR "digital health"[Title/Abstract] OR "remote monitoring"[Title/Abstract] OR "mobile applications"[MeSH Terms] OR "wearable devices"[MeSH Terms] OR "internet"[MeSH Terms] OR "social media"[MeSH Terms] OR "virtual reality"[MeSH Terms] OR "remote consultation"[MeSH Terms])  **AND**  ("Diabetes Mellitus"[MeSH Terms] OR "Diabetes Mellitus, Type 1"[MeSH Terms] OR "Diabetes Mellitus, Type 2"[MeSH Terms] OR "Hypertension"[MeSH Terms] OR "Essential Hypertension"[MeSH Terms] OR "Cardiovascular Diseases"[MeSH Terms] OR "Heart Diseases"[MeSH Terms] OR "Stroke"[MeSH Terms] OR "Metabolic Syndrome"[MeSH Terms] OR "Obesity"[MeSH Terms] OR "Cholesterol"[MeSH Terms] OR "Triglycerides"[MeSH Terms] OR "Physical Activity"[MeSH Terms] OR "Exercise"[MeSH Terms] OR "Diet"[MeSH Terms])  **AND**  ("rural population"[MeSH Terms] OR "rural health"[MeSH Terms] OR "remote"[Title/Abstract] OR "regional"[Title/Abstract] OR "Australia"[MeSH Terms] OR "Australian Capital Territory"[Title/Abstract] OR "Northern Territory"[Title/Abstract] OR "Queensland"[Title/Abstract] OR "New South Wales"[Title/Abstract] OR "South Australia"[Title/Abstract] OR "Western Australia"[Title/Abstract] OR "Tasmania"[Title/Abstract]) |
| **MEDLINE (Ovid)** | “Mobile Applications” OR “mHealth.mp.” OR “mobile health.mp.” OR “ehealth.m p.” OR “telemedicine/ or distance counseling” OR “mental” OR “electronic health records” OR  “health information exchange” OR “health apps.mp.” OR “wearable electronic devices/ or fitness trackers” OR “hearing aids” or smart glasses” OR “Remote Sensing Technology” OR “internet/ OR “internet-based intervention” OR “social” OR “Media” OR “virtual care.mp.” OR “digital coaching.mp.” OR “tele coaching.mp.” OR “digital therapeutics.mp.” OR “technology-based health interventions.mp.” OR “wearable technology.mp.” OR “digital health technology.mp.” OR “Remote Consultation” OR “tele intensive care.mp.” OR “virtual reality/ or avatar/ or haptic technology” OR “Videoconferencing” OR “Cell Phone/ or 5martphone” OR “phone call.mp.” OR “("Digital Health" or "Digital health technology" OR "Digital technology" OR "Digital technologies" OR "Digital health technologies" OR "Health technology" OR "Health technologies" or "Health information technology" or "Health information technologies" OR "Digital therapeutics" OR "educational technology" OR "educational technologies").mp. [mp=title, book title, abstract, original title, name of substance word, subject heading word, floating sub-heading word, keyword heading word, organism supplementary concept word, protocol supplementary concept word, rare disease supplementary concept word, unique”  **AND**  “Metabolic Syndrome” OR “Obesity, Abdominal” OR “diabetes mellitus, type 2/ or diabetes mellitus” OR “lipoatrophic” OR “hypertension/ or essential hypertension” OR “hypertension, malignant” OR “hypertension” OR crisis” OR “hypertensive retinopathy” OR “masked hypertension” OR “white coat hypertension” OR “cardiometabolic risk factors/ or obesity paradox” OR “Triglycerides” OR “fasting glucose.mp.” OR “Blood Glucose” OR “("Lifestyle behav*" or "Blood pressure" or "BP" or "cholesterol levels" or 'triglycerid* or "fasting glucose" or 'weight' or 'BMI' or "waist circumference" or "heart rate" or "lipid profile" or "cardiometab*" or 'diabet*' or 'hypertension' or "cardiovascular health" or 'obesity' or "metabolic syndrome").mp. [mp=title, book title, abstract, original title, name of substance word, subject heading word, floating sub-heading word, keyword heading word, organism supplementary concept word, protocol supplementary concept word, rare disease supplementary concept word, unique identifier, synonyms, population supplementary concept word, anatomy supplementary concept word]”  **AND**  (rural.mp. OR remote.mp. OR regional.mp. OR suburban.mp. OR Australia.mp. OR "Australian Capital Territory".mp. OR "New South Wales".mp. OR "Victoria".mp. OR "Queensland".mp. OR "Tasmania".mp. OR "South Australia".mp. OR "Western Australia".mp. OR "Northern Territory".mp.) |
| **EMBASE (Ovid)** | ('telemedicine'/exp OR 'telehealth'/exp OR 'ehealth'/exp OR 'mhealth'/exp OR 'mobile application'/exp OR 'wearable device'/exp OR 'internet intervention'/exp OR 'digital health'/exp OR 'remote consultation'/exp OR 'virtual reality'/exp OR 'social media'/exp OR 'email'/exp OR 'internet access'/exp OR 'internet'/exp OR 'digital therapeutic'/exp OR 'digital coaching'/exp OR 'remote monitoring'/exp OR 'avatar'/exp)  **AND**  ('cardiometabolic risk'/exp OR 'cardiovascular disease'/exp OR 'cardiovascular risk'/exp OR 'lifestyle behavior'/exp OR 'hypertension'/exp OR 'essential hypertension'/exp OR 'masked hypertension'/exp OR 'pregnancy induced hypertension'/exp OR 'pulmonary hypertension'/exp OR 'renal hypertension'/exp OR 'metabolic syndrome x'/exp OR 'obesity'/exp OR 'pediatric obesity'/exp OR 'cholesterol'/exp OR 'hdl cholesterol'/exp OR 'ldl cholesterol'/exp OR 'triglyceride'/exp OR 'heart disease'/exp OR 'diabetes mellitus'/exp OR 'diabetes mellitus type 1'/exp OR 'diabetes mellitus type 2'/exp OR 'diabetic cardiomyopathy'/exp OR 'diabetic neuropathy'/exp OR 'physical activity'/exp OR 'exercise'/exp OR 'diet'/exp)  **AND**  ('rural area'/exp OR 'remote area'/exp OR 'regional health service'/exp OR  'suburban area'/exp OR 'rural population'/exp OR 'regional population'/exp OR  'australia'/exp OR 'australian capital territory'/exp OR 'northern territory'/exp OR  'queensland'/exp OR 'south australia'/exp) |
| **Scopus** | ((telehealth OR telemedicine OR "mobile health" OR mHealth OR "eHealth" OR "digital health" OR "digital intervention" OR "remote monitoring" OR "remote patient monitoring" OR "wearable technology" OR "wearable device" OR "smartphone application" OR "mobile application" OR "virtual care" OR telemonitoring OR "SMS intervention" OR "text messaging"))  AND  ((diabetes OR "type 2 diabetes" OR "cardiovascular disease" OR "cardiovascular diseases" OR CVD OR "heart disease" OR hypertension OR "high blood pressure" OR obesity OR overweight OR stroke OR "metabolic syndrome" OR hyperlipidemia OR dyslipidemia OR atherosclerosis))  AND  ((rural OR remote OR regional OR underserved OR "non-urban" OR "rural health" OR "rural health services" OR "remote communities" OR "isolated communities" OR "sparsely populated areas" OR "outback" OR "rural Australia")) |
| **CINAHL (EBSCOhost)** | ((MH "Mobile Applications") OR (MH "Multimedia") OR (MH "Internet-Based Intervention") OR (MH "Social Media") OR (MH "Email") OR (MH "Internet Access") OR (MH "Internet") OR "digital therap*" OR "digital coach*" OR (MH "Virtual Reality Exposure Therapy") OR (MH "Virtual Reality") OR (MH "Avatars") OR "online health platform*" OR (MH "Remote Consultation") OR (MH "Telepathology") OR (MH "Teleradiology") OR (MH "Telerehabilitation") OR "Wearable devic*" OR "health app*" OR "ehealth" OR "mhealth" OR (MH "Telehealth") OR "telehealth" OR (MH "Digital Health") OR (MH "Individualized Medicine") OR (MH "Wearable Sensors"))  AND  ((MH "Cardiometabolic Risk Factors") OR (MH "Cardiovascular Risk Factors") OR "Lifestyle behavio*" OR (MH "Hypertension") OR (MH "Essential Hypertension") OR (MH "Masked Hypertension") OR (MH "Pregnancy-Induced Hypertension") OR (MH "Hypertension, Isolated Systolic") OR (MH "Hypertension, Malignant") OR (MH "Hypertension, Portal") OR (MH "Hypertension, Pulmonary") OR (MH "Hypertension, Refractory") OR (MH "Hypertension, Renal") OR (MH "Hypertension, White Coat") OR "cholesterol" OR (MH "Lipoproteins, HDL Cholesterol") OR (MH "Lipoproteins, LDL Cholesterol") OR (MH "Metabolic Syndrome X") OR "metabolic syndrome" OR (MH "Triglycerides") OR (MH "Obesity") OR (MH "Obesity Paradox") OR (MH "Obesity, Maternal") OR (MH "Obesity, Morbid") OR (MH "Pediatric Obesity") OR (MH "Prader-Willi Syndrome") OR (MH "Pickwickian Syndrome") OR (MH "Cardiovascular Diseases+") OR (MH "Cardiovascular Abnormalities") OR (MH "Heart Diseases") OR (MH "Diabetes Mellitus") OR (MH "Diabetes Mellitus, Type 1") OR (MH "Diabetes Mellitus, Type 2") OR (MH "Diabetic Cardiomyopathies") OR (MH "Diabetic Coma") OR (MH "Diabetic Ketoacidosis") OR (MH "Diabetic Nephropathies") OR (MH "Diabetic Neuropathies") OR (MH "Donohue Syndrome") OR (MH "Maturity-Onset Diabetes of the Young") OR (MH "Pregnancy in Diabetes") OR (MH "Sports") OR (MH "Physical Activity") OR (MH "Leisure Activities") OR (MH "Physical Performance") OR (MH "Diet+"))  AND  ((MH "Suburban Population") OR (MH "Rural Population") OR (MH "Regional Area") OR (MH "Rural Areas") OR (MH "Suburban Areas") OR (MH "Australia") OR (MH "Australian Capital Territory") OR (MH "Northern Territory") OR (MH "Queensland") OR (MH "South Australia")) |
