# Supplementary material for: Digital Health Interventions for Cardiometabolic Health Outcomes in Rural and Remote Australia: A Systematic Review
Source: Aust J Rural Health. 2025 Dec 26;33(6):e70130. doi: 10.1111/ajr.70130 (PMC12742273; doi:10.1111/ajr.70130)
Supplement: Supplementary file 1 — File S1: PRISMA 2020 checklist showing compliance with reporting guidelines for this systematic review. File S2: Full electronic search strategies for included databases (PubMed, Embase, Ovid MEDLINE and CINAHL). File S3: Characteristics of the included studies. File S4: Describe summary of study characteristics and health outcomes from digital health intervention trials addressing cardiometabolic conditions in rural and remote settings. File S4: Risk‐of‐bias assessment for all included randomised controlled trials across the five RoB 2 domains and overall bias rating. [file AJR-33-0-s001.zip › ajr70130-sup-0004-supinfo03.docx]

Supplementation file 3: Characteristics of the included studies

| **Author and Publication years** | **Country and Study Setting** | **Study design** | **Sample size/description** | **Intervention period** | **Method** | **Intervention/types** |
| --- | --- | --- | --- | --- | --- | --- |
| Adu et al., 2020 | North Queensland, Australia. | The study employed a sequential explanatory mixed-methods design, in which quantitative survey data were collected and analysed first, followed by qualitative interviews to further explore and explain the quantitative findings | A total of 4,984 patients were invited via email.  Of these, 79 patients (1.59%) completed the eligibility form. Sixty-seven were deemed eligible and granted access to the app; however, 17 did not log in.  The final enrolled sample consisted of 50 participants, with 41 completing the study per protocol, resulting in a retention rate of 82%.  Seventeen participants also took part in follow-up qualitative interviews. | Three weeks | As part of the study, participants were given access to the *My Care Hub* mobile health (mHealth) application for a period of three weeks to assist with self-management of diabetes. | The study assessed app retention and engagement using the Frequency, Intensity, Time, and Type (FITT) framework.  Retention rate:   - 82% of participants (41 out of 50) completed the study.   Engagement:   - 84% of participants (42 out of 50) used the app at least once. - Average app usage across three weeks was as follows:  • Week 1: 5.2 days  • Week 2: 4.8 days  • Week 3: 3.8 days - The most commonly used most used feature was blood glucose level (BGL) tracking (68% of users), while the least used feature was carbohydrate tracking (2% of users). - 57% of users opened push notifications within 24 hours.   Participant feedback (qualitative findings) indicated that the app:   - Improved awareness of BGL levels - Encouraged accountability in diabetes self-management - Provided motivation through graphical tracking features - Some users expressed a desire for automated data input (e.g., Bluetooth integration for BGL logs)   Others wanted more detailed historical data analysis and feedback on physical activity |
| Champion et al., 2022 | Participants were recruited from regional, rural, and remote communities across South Australia | A prospective cohort design was used to compare cardiac rehabilitation (CR) utilisation and associated clinical outcomes prior to and during the COVID-19 pandemic | A total of 1,954 patients were included in the cohort study: – 922 in the pre-COVID-19 period (February to June 2019) – 1,032 in the during COVID-19 period (February to June 2020) | 48 weeks | This study employed a mixed-methods multi-design approach, combining a prospective cohort study with a cross-sectional survey. The prospective cohort component was used to compare cardiac rehabilitation (CR) utilisation and clinical outcomes among patients referred to centre-based and telehealth CR programs before and during the COVID-19 pandemic across regional and rural South Australia. The cross-sectional survey component was conducted to assess the experiences of regional and rural CR services in response to the pandemic and the rapid adoption of telehealth delivery models. | - Intervention was cardiac rehabilitation (CR) delivered through two modes: Centre-based CR, comprising traditional in-person group and individual sessions - Telehealth-based CR, involving remote delivery via telephone, with optional video consultations - During the COVID-19 period, centre-based services rapidly transitioned to telehealth delivery, replacing group sessions with telephone-based CR. |
| Drew et al., 2022 | The study was conducted in Newcastle and the surrounding urban and suburban regions of New South Wales, Australia | Randomized Controlled Trial (RCT) | Age=18-70, n=125 Male | 24 weeks | RCT on SHERIT PROGRAM | The SHED-IT: Recharge program (*Self-Help, Exercise and Diet using Information Technology*) is a lifestyle intervention designed to support men's physical and mental health through self-directed resources delivered via digital platforms. |
| Duncan et al., 2020 | Urban in Newcastle area, New South Wales, Australia | A three-arm randomised controlled trial (RCT) | n=116, F=82, M=34, Age+ 18-65 | The study duration was 48 weeks, with the primary endpoint assessed at 24 weeks and final follow-up at 48 weeks. | Three-arm randomised controlled trial (RCT) | Participants in both the Enhanced and Traditional groups were provided with personalised dietary recommendations and given access to the *Balanced* smartphone application, along with an additional calorie-counting platform (*CalorieKing Wellness Solutions Inc.*, La Mesa, CA, USA). They also received a set of body weight scales (*Tanita HD-380*), a Fitbit activity tracker (*Fitbit Alta*), and a participant handbook.  The intervention incorporated behaviour change techniques, such as education, goal setting, self-monitoring, and behavioural feedback, to operationalise constructs from social cognitive and self-regulation theories tailored to the target behaviours. |
| Eakin et al., 2010 | Queensland, Australia – specifically in Logan, a large urban centre with a diverse ethnic and socio-economic population. | Randomized Controlled Trial (RCT) | A total of 300 participants, aged between 20 and 75 years and diagnosed with type 2 diabetes, were recruited from 10 general practices using electronic medical record searches | A 72-week intervention with follow-up at 96 weeks | Participants were randomised to a 72-week telephone-delivered weight loss intervention focused on physical activity, diet, and behavioural therapy, or to usual care. Follow-up assessments were conducted at 24, 72, and 96 weeks | The telephone-delivered program comprised a 24 week intensive phase involving 15 calls, followed by 48 weeks maintenance phase with 48 weekly calls. The intervention was underpinned by Social Cognitive Theory and targeted weight loss, physical activity, and glycaemic control |
| Fatehi et al., 2013 | Remote and rural cities in Queensland | Cross-sectional study | 56 (M=31, F=25, T1D=18, T2D=37, Other DM=1) | 20 weeks | questionnaire based consultation | Video conference consultations focused on insulin dose adjustments and allied health support |
| Graham et al., 2024 | The study was conducted in rural and remote regions of South Australia and the NSW border area, specifically Broken Hill | Exploratory qualitative study | The study included 11 participants (8 males and 3 females), with a mean age of 53.8 years and an age range of 44 to 64 years | November 5, 2020 – June 30, 2022 (72 weeks) | A video-based telehealth service was used for the management of diabetic foot disease (DFD). One-on-one semi-structured interviews were conducted and analysed using thematic analysis | The intervention involved a real-time, video-based telehealth service designed to support the management of diabetes-related foot disease (DFD) |
| Goh et al., 2023 | Regional and rural setting in South Australia. | Retrospective cohort study | The study included 189 stroke patients, comprising 31 in the pre-intervention group and 158 in the post-intervention group, with comparable baseline characteristics between the cohorts. | Data were collected over two periods: pre-implementation (1 December 2017 to 3 June 2018; 24 weeks) and post-implementation (4 June 2018 to 31 December 2019; 72 weeks). | A telestroke service was implemented to provide 24/7 telehealth access to stroke neurologists for hyperacute stroke management, supported by local education initiatives and enhanced ambulance triage protocols. | The telestroke service included teleconsultations via secure video conferencing, supported by multimodal CT perfusion imaging and the implementation of comprehensive stroke management plans for hyperacute stroke care. |
| Goode et al., 2015 | The study was conducted in primary care practices situated in a disadvantaged urban community in Logan, Queensland, approximately 35 kilometres south of Brisbane | Randomized Controlled Trial (RCT) | A total of 151 participants were allocated to the intervention arm. All were adults aged between 20 and 75 years, diagnosed with type 2 diabetes, and residing in a socioeconomically disadvantaged area | 72 weeks | Intervention dose was categorised into tertiles: low (<12 calls), medium (12–20 calls), and high (≥21 calls) outcomes were measured at baseline, 24, 72, and 96 weeks using objective tools (e.g., accelerometers for moderate-to-vigorous physical activity [MVPA]) and validated questionnaires | Participants received telephone counselling for lifestyle-based weight loss over 72 weeks period, involving up to 27 calls focused on physical activity, dietary habits, and behaviour change strategies |
| Lombard et al., 2016 | Location: 41 rural towns in Victoria, Australia Distance: Within a 100 to 400 km radius from Melbourne | Randomised Controlled Trial (RCT) | Total participants: 649 women Clusters: 41 rural towns (21 intervention, 20 control)  Mean age: 39.6 years (±6.7) Age range: 18 to 50 years (reproductive-aged women) | Follow-up at 48 weeks:   - 76.2% retention in the intervention group - 78.2% retention in the control group | Intervention Group   - One 60-minute group session: Interactive and facilitator-led, featuring five simple health messages (e.g., increase fruit and vegetable intake, walk daily) - One program manual: Self-guided behavioural activities - Monthly SMS text messages: Twelve messages reinforcing key behaviours - One 20-minute phone coaching call at 12 weeks   Control Group   - One 45-minute general health education session: Non-interactive - Topics: General women’s health, Australian dietary and physical activity guidelines - No further contact | The intervention was a low-intensity, multi-component lifestyle program aimed at preventing weight gain in rural women. It included:   1. Group Session: One 60-minute interactive session delivering simple health messages and self-management skills 2. Program Manual: A take-home manual to guide goal setting, self-monitoring, and action planning 3. SMS Text Messages: Monthly personalised messages reinforcing healthy behaviours 4. Phone Coaching: A 20-minute call at week 12 using motivational interviewing to support behaviour change |
| Mullan et al., 2022 | Western Queensland, Australia (WQPHN – Western Queensland Primary Health Network) | Quasi-experimental study comparing VCDEP practices with non-VCDEP practices  Pre-post intervention study measuring diabetes management indicators at two time points: October 2019 and March 2021. | VCDEP Practices: 1,369 diabetes patients in October 2019, increasing to 1,518 in March 2021  Non-VCDEP Practices: 939 diabetes patients in October 2019, increasing to 1,005 in March 2021  Total patients across both groups in 2021: 2,523 | 24 weeks | Data source: De-identified patient data extracted from general practices via the Practice Aggregation Tool for the Clinical Audit Tool (PATCAT) platform by PenCS  Data collection: Monthly reports extracted from practices via WQPHN's data platform | The intervention is the Virtual Care Diabetes Education Program (VCDEP), delivered across multiple locations and primary care practices. It provides chronic disease education and diabetes self-management support through Credentialled Diabetes Educators (CDEs). Data are collected and analysed to evaluate program effectiveness. |
| Sangster et al., 2015 | Setting: Both urban and rural Australia Participant recruitment: From two urban hospitals in Sydney and two rural hospitals located approximately 200 km and 450 km south-west of Sydney | Randomised Controlled Trial (RCT) | R=140 | 32 weeks | Cost-effectiveness analyses compare a ‘‘new’’ | Participants received mailed brochures, a calendar (to record nutrition and physical activity goals), and a pedometer. They took part in four telephone-coaching and goal-setting sessions on weight, nutrition and physical activity over an eight-week period, plus two booster calls after the intervention |
| Smith et al., 2003 | Rural and remote areas of Queensland | A retrospective descriptive review | n=160, 10=education session, 28 | 112 weeks | Consultations deliveries were delivered via videoconference using standard commercial videoconferencing equipment (Sony models 5100P and 1500) | A total of 160 patient consultations and 10 education sessions were delivered via videoconference during the 28-month study period |
| Talay et al., 2025 | Rural/remote Australia (MMM 3–6, regional towns to remote communities) | Rural/remote Australia (MMM 3–6, regional towns to remote communities) | 32 (19 M, 13 F); mean age 45.8 yrs; mean BMI 34.5 kg/m² | ≥3 weeks of program use before interview  Ongoing subscription model | COREQ; phone interviews; Braun & Clarke thematic analysis | Digital Weight-Loss Service (DWLS): GLP-1 RA medication + lifestyle coaching + MDT care via app (synchronous & asynchronous consults) |
| Tually et al., 2003 | Kalgoorlie, Western Australia, a remote town 700 km east of Perth | Preliminary feasibility study | 42 patients (median age: 58 years, range 29–73; 21 males) referred for exclusion of acute coronary syndrome | June to August 2002 (12 weeks) | Myocardial perfusion scintigraphy (MPS) data were processed on-site using a portable nuclear medicine system. JPEG images were transmitted to a secure web server and reviewed by referring clinicians and cardiologists in Perth. | Web-based telemedicine intervention enabling group consultations and rapid access to MPS results to support clinical decision-making |
| Warren et al., 2017 | Townsville area (urban), Queensland, Australia | Randomised Controlled Trial (RCT) | Participants:126 (63 in the intervention arm and 63 in the control arm).  Eligibility criteria: Adults aged years ≥18 with type 2 diabetes, HbA1c ≥ 58 mmol/mol (7.5%), and stable comorbidities | 24 weeks | Participants in the intervention group received telemonitoring devices, regular consultations, and personalised care plans managed by diabetes care coordinators. Data collection included biomedical measures, quality of life (SF-12), and healthcare costs | - Telemonitoring for type 2 diabetes management, including home monitoring devices for blood glucose and blood pressure, with support from a diabetes care coordinator. |
| Williams M., 2020 | Setting: Rural paediatric diabetes telehealth clinics at four rural hospitals (Proserpine, Bowen, Clermont, and Moranbah) in Queensland, Australia | Retrospective audit and survey study | Participants: 19 children and adolescents with type 1 diabetes mellitus (T1DM) | 48 weeks | - Face-to-face and telephone surveys with patients, parents, and healthcare professionals (HCPs) - Retrospective audit of patient records over 12 months - Thematic analysis of qualitative feedback | Telehealth-based diabetes management for paediatric patients in rural settings  Key components of the intervention:   - Videoconferencing consultations between rural patients and specialists at Mackay Base Hospital (MBH) - Multidisciplinary approach: A paediatrician, diabetes nurse educator (DNE), dietitian, social worker, and psychologist participated in consultations - Local healthcare support: A DNE or a trained nurse was present with the child and parent at the rural site. - Point-of-care HbA1c testing using a portable analyser at the rural sites - Remote insulin pump and glucose monitor downloads to assist in treatment decisions. Electronic prescriptions sent directly to pharmacies |
