# Supplementary material for: Digital Health Interventions for Cardiometabolic Health Outcomes in Rural and Remote Australia: A Systematic Review
Source: Aust J Rural Health. 2025 Dec 26;33(6):e70130. doi: 10.1111/ajr.70130 (PMC12742273; doi:10.1111/ajr.70130)
Supplement: Supplementary file 1 — File S1: PRISMA 2020 checklist showing compliance with reporting guidelines for this systematic review. File S2: Full electronic search strategies for included databases (PubMed, Embase, Ovid MEDLINE and CINAHL). File S3: Characteristics of the included studies. File S4: Describe summary of study characteristics and health outcomes from digital health intervention trials addressing cardiometabolic conditions in rural and remote settings. File S4: Risk‐of‐bias assessment for all included randomised controlled trials across the five RoB 2 domains and overall bias rating. [file AJR-33-0-s001.zip › ajr70130-sup-0005-supinfo04.docx]

**Supplementation file 4**: Describe summary of Study Characteristics and Health Outcomes from Digital Health Intervention Trials Addressing Cardiometabolic Conditions in Rural and Remote Settings

| **Author** | **Diabetes mellitus outcome** | **Cardiovascular disease outcome** | **Hypertension and Stroke outcome** | **Obesity outcome** |
| --- | --- | --- | --- | --- |
| Adu et al., 2020 | - Changes in the intervention group Retention rate: 84% (42/50 participants adhered to the study protocol) - App usage: Average use of 11 out of 14 days during the first two weeks - Continued engagement:   - 85% (36/42) continued after Week 1.   - 71% (30/42) continued into Week 3 - Most frequently used features:   - Blood glucose tracking (68%)   - Access to educational content (13%) - Push notification engagement: 57% (24/42) opened messages within 24 hours   Reported behavioural changes (qualitative findings)   - Improved self-care efforts (blood glucose tracking, exercise, diet monitoring) - Increased diabetes awareness (visualisation of blood glucose trends) - Higher accountability (data logging prompted more conscious management) | Not reported in the primary study | Not reported in the primary study | Not reported in the primary study |
| Champion et al., 2022 | Not reported in the primary study | Intervention Outcomes: Cardiac Rehabilitation (CR) Before vs During COVID-19  Primary Outcomes:   - CR attendance was significantly lower during COVID-19 (46.6%) compared with pre-COVID-19 (59.9%) *(P < 0.001)* - CR completion was also significantly lower during COVID-19 (42.4%) compared with pre-COVID-19 (75.4%) *(P < 0.001)*   Secondary outcomes:   - No significant difference in waiting time to start CR between the two periods - Clinical outcomes, including cardiovascular-related readmissions and deaths, were similar before and during COVID-19 - Telehealth CR programs showed higher attendance and completion rates than centre-based CR   Statistical significance of intervention effect:   - Lower CR uptake during COVID-19:   - SHR = 0.77 (95% CI: 0.68–0.87) for CR attendance   - SHR = 0.66 (95% CI: 0.58–0.76) for CR completion - Mode of delivery mattered:   - Telehealth delivery was associated with higher CR attendance and completion compared to centre-based programs - No significant effect on clinical outcomes (CV readmissions and deaths) | Not reported in the primary study | Not reported in the primary study |
| Drew et al., 2022 | Change in weight (intervention vs control)   - At 3 months (post-intervention):   - Mean difference: –4.2 kg (95% CI: –5.6 to –2.8), *P* < 0.001 - At 6 months (follow-up):   - Mean difference: –3.9 kg (95% CI: –5.4 to –2.5), *P* < 0.001   Summary Significant and sustained weight loss was observed in the intervention group compared with the control group | Not reported in the primary study | Not reported in the primary study | Not reported in the primary study |
| Duncan et al., 2020 | Not reported in the primary study | Not reported in the primary study | Not reported in the primary study | Weight change (intervention vs control)   - After 6 months: Mean difference = –0.92 kg (95% CI: –3.33 to 1.48), *P* > 0.05 (not significant) - After 12 months: Mean difference = 0.00 kg (95% CI: –2.62 to 2.62), *P* > 0.05 (not significant)   Waist circumference (intervention vs control)   - After 6 months: Mean difference = –0.62 cm (95% CI: –3.18 to 1.95), *P* > 0.05 (not significant) - After 12 months: Mean difference = 0.59 cm (95% CI: –2.17 to 3.36), *P* > 0.05 (not significant) |
| Eakin et al., 2010 | Telehealth model Portable nuclear medicine (NM) system with internet-based remote consultation for cardiac care  Clinical impact   - 76% of patients had treatment plans altered based on telehealth-assisted diagnosis - 50% of scans showed abnormal myocardial perfusion - 5% of patients required urgent medical intervention   Reported benefits   - Earlier risk stratification - Improved access to expert cardiology input in a remote setting - Avoidance of unnecessary hospital admissions and investigations | Not reported in the primary study | Not reported in the primary study | Not reported in the primary study |
| Fatehi et al., 2013 | **Consultation outcomes**   - 62% of consultations resulted in medication changes:   - Insulin dose adjustments: 39%   - Initiation of new medications: 4%   - Cessation or regimen changes: 7% - 75% of consultations included laboratory test requests - 86% of patients had follow-up appointments scheduled   **Support during telehealth**   - Nurse support present in 66% of consultations | Not reported in the primary study | Not reported in the primary study | Not reported in the primary study |
| Graham et al., 2023 | The study utilised real-time, video-based telehealth services to manage diabetic foot disease (DFD) in rural and remote Aboriginal and Torres Strait Islander communities. patients experienced reduced travel burdens, improved access to specialists, and enhanced communication. Participants reported feeling reassured that specialists had seen their feet, which positively influenced their emotional wellbeing, trust, and participation in care.  The telehealth model facilitated access to multidisciplinary treatment and strengthened patient-centred enabling collaboration between local health professionals, community members, and specialists during consultations. This approach addressed practical barriers such as travel and limited access to facilities, while also improving cultural safety and patient engagement. These findings underscore the potential of telehealth to care quality and outcomes for Indigenous patients with DFD, particularly in underserved regions. | Not reported in the primary study | Not reported in the primary study | Not reported in the primary study |
| Goh et al., 2023 | Not reported in the primary study | Not reported in the primary study | - Mortality reduction (pre- vs post-intervention) at 6 months:   - Hazard Ratio (HR) = 0.53   - 95% CI: 0.41–0.69   - *P*< 0.001 - At 12 months:   - HR = 0.58   - 95% CI: 0.44–0.76   - *P*< 0.001 - Composite care quality score improvement Selected indicators:   - 95% CI: 0.004–0.134   - *P* = 0.04 - All indicators:   - 95% CI: 0.005–0.140   - *P* = 0.03 | Not reported in the primary study |
| Goode et al., 2015 | Intervention vs Control  Weight Loss   - At 6 months:   - High-call group: –2.7 kg (95% CI: –4.0 to –1.5), *P* < 0.001   - Medium-call group: –0.9 kg (95% CI: –2.3 to 0.4), *P* = 0.18   - Low-call group: No significant change, *P* = 0.80 - At 18 months:   - High-call group: –2.5 kg (95% CI: –3.8 to –1.3), *P* < 0.001   - Medium-call group: –1.0 kg (95% CI: –2.5 to 0.5), *P* = 0.18   - Low-call group: No significant change, *P* = 0.19 - At 24 months:   - High-call group: –2.0 kg (95% CI: –3.3 to –0.7), *P* = 0.002   - Medium-call group: –1.2 kg (95% CI: –2.8 to –0.3), *P* = 0.046   - Low-call group: No significant change, *P* = 0.29 - Moderate to vigorous physical activity (MVPA) - At 6 months:   - High-call group: RR = 1.48 (95% CI: 1.08 to 2.04), *P* = 0.02   - Medium-call group: RR = 1.22 (95% CI: 0.86 to 1.72), *P* = 0.27   - Low-call group: No significant change, *P* = 0.56 - At 18 and 24 months: No significant changes in any group - Glycaemic control (HbA1c) High-call group: Small reduction at 6 months   - RR = 0.96 (95% CI: 0.92 to 0.99), *P* = 0.02,   - Not sustained at later time points   Medium- and low-call groups: No significant changes across all time points. | Not reported in the primary study | Not reported in the primary study | Not reported in the primary study |
| Lombard et al., 2016 | Not reported in the primary study | Not reported in the primary study | Not reported in the primary study | The intervention led to a significant prevention of weight gain over one year:   - Mean weight change:   - Intervention group: –0.48 kg (95% CI: –0.99 to 0.03)   - Control group: +0.44 kg (95% CI: –0.09 to 0.97) - Between-group difference (adjusted):   - –0.87 kg (95% CI: –1.62 to –0.13), *P* = 0.02 |
| Mullan et al., 2022 | Improvements in VCDEP Practices   - HbA1c recording increased from 72.2% to 77.7% (P < 0.001) - eGFR recording increased from 76.6% to 81.6% (P < 0.001) - Total cholesterol recording increased from 73.3% to 77.0% (P = 0.022) - Foot examination recording increased from 6.4% to 8.8% (P = 0.015) - Microalbuminuria target achievement was higher in VCDEP practices (58.8% vs. 51.3%, P = 0.006)   No Significant Changes in Meeting Clinical Targets   - Blood pressure control (<140/90 mmHg) slightly decreased from 69.1% to 66.4% (P = 0.205) - HbA1c control (<7%) slightly decreased from 48.7% to 46.5% (P p = 0.306) - eGFR in target (>90 mL/min/1.73 m²) slightly decreased from 38.5% to 36.7% (P = 0.394)   BMI in target (<25 kg/m²) remained low, from 10.1% to 10.7% (P = 0.672) | Not reported in the primary study | Not reported in the primary study | Not reported in the primary study |
| Sangster et al., 2015 | Not reported in the primary study | Weight Loss:   - Healthy Weight group lost more weight than the Physical Activity group (*P* = 0.005) - BMI ≥ 25subgroup: Mean weight loss of 1.6 kg (Healthy Weight) vs 0.4 kg (Physical Activity) - 19% of Healthy Weight participants lost ≥4.0 kg (5% body weight) vs 11% in Physical Activity group   Quality of Life Improvements:   - The Healthy Weight group had a greater improvement in quality of life (0.081 QALYs vs 0.037 QALYs, *P < 0.05)* - The Healthy Weight group started with a lower baseline quality of life but had greater improvement over time   Cost-effectiveness:   - Healthy Weight intervention was both cost-saving and more effective than the Physical Activity intervention - Overall cost per participant:   - Healthy Weight: $1,260   - Physical Activity: $2,112 - Incremental QALYs gained:   - Healthy Weight: 0.034 QALYs   - Physical Activity: 0.018 QALYs - Healthy Weight was cost-effective for rural participants and those who did not attend CR - Less effective but cost-saving for urban participants and those who attended CR   Health Care utilisation:   - Physical Activity group had more specialist visits and hospital admissions in the short and medium term   Higher exercise-related expenses in the Physical Activity group ($60.85 vs $15.21, *P = 0.003)* | Not reported in the primary study | Not reported in the primary study |
| Smith et al., 2003 | **Service delivery**:   - 160 patient consultations and 10 education sessions over 28 months - 21 routine clinics conducted (76% focused on diabetes) - Clinics involved 2–14 patients per session (median = 6 patients)   **Session durations**:   - Routine clinics: Median 130 minutes (IQR 60–240) - Single-patient consultations: Median 45 minutes (IQR 30–60) - Education sessions: 60–90 minutes   **Overall telehealth utilisation**:   - 5,080minutes of videoconferencing across 56 sessions   Reported benefits or outcomes:   - Improved access to multidisciplinary care (specialists, nurse educators, dietitians, mental health workers) - Reduced travel burden for patients and healthcare providers   Increased support for families in remote areas through tailored education sessions | Not reported in the primary study | Not reported in the primary study | Not reported in the primary study |
| Talay et al., 2025 | Not reported in the primary study | Not reported in the primary study | Not reported in the primary study | The study utilised a digital health intervention (DHI) classified primarily under *telemedicine and tele-prescription models*, with elements of self-help tools and integrated care. The Eucalyptus Australia DWLS combined app-based consultations, remote prescribing of GLP-1 RA medication, lifestyle coaching, and multidisciplinary team support. The DHI facilitated access to continuous obesity care for regional Australians by reducing unmanageable travel barriers, improving care continuity, and enhancing patient confidence and motivation through early weight loss. However, limitations were noted, including inadequate app functionality and the tendency of some patients to use the service primarily for medication access rather than lifestyle change. |
| Tually et al., 2003 | Not reported in the primary study | Telehealth model Portable nuclear medicine (NM) system with internet-based remote consultation for cardiac care  Clinical impact   - 76% of patients had treatment plans altered based on telehealth-assisted diagnosis - 50% of scans showed abnormal myocardial perfusion - 5% of patients required urgent medical intervention   Reported benefits   - Earlier risk stratification - Improved access to expert cardiology input in a remote setting   Avoidance of unnecessary hospital admissions and investigations | Not reported in the primary study | Not reported in the primary study |
| Warren et al., 2017 | - Glycaemic Control (HbA1c): Intervention group: Median HbA1c decreased from 8.4% to 7.5% - Control group: Median HbA1c remained unchanged at 8.1% (65 mmol/mol) - *P = 0.004*   Cost**-**effectiveness**:**   - Total mean cost:   - Intervention group: $3,781   - Control group: $4,662   - *P < 0.001* | Not reported in the primary study | Not reported in the primary study | Not reported in the primary study |
| Williams M., 2020 | **Intervention group comparison: central clinic vs telehealth**   - **HbA1c levels:**   - Telehealth group: Mean HbA1c = 7.5%   - Central clinic group: Mean HbA1c = 8.4%   - No P value reported; results suggest non-inferiority of telehealth care in glycaemic control - **Travel burden:**   Telehealth care reduced the need for long-distance travel, avoiding trips of 120–280 km for participants | Not reported in the primary study | Not reported in the primary study | Not reported in the primary study |
